# Clinical characteristics and outcomes in COVID-19 in kidney transplant recipients: a propensity score matched cohort study

**DOI:** 10.3389/fmed.2024.1350657

**Published:** 2024-04-15

**Authors:** Polianna Delfino-Pereira, Vanessa das Graças José Ventura, Magda Carvalho Pires, Daniela Ponce, Gabriel Assis Lopes do Carmo, Lilian Pires de Freitas do Carmo, Bruno Barbosa Miranda de Paiva, Alexandre Vargas Schwarzbold, Angélica Gomides dos Reis Gomes, Bruno Mateus de Castro, Carísi Anne Polanczyk, Christiane Corrêa Rodrigues Cimini, Daniela Antunes de Lima, Fabiano Carvalho de Sousa, Frederico Bartolazzi, Giovanna Grunewald Vietta, Heloisa Reniers Vianna, José Miguel Chatkin, Karen Brasil Ruschel, Luciane Kopittke, Luís César de Castro, Marcelo Carneiro, Priscilla Pereira dos Reis, Milena Soriano Marcolino

**Affiliations:** ^1^Medical School, Universidade Federal de Minas Gerais, Belo Horizonte, Brazil; ^2^Institute for Health Technology Assessment (IATS), Porto Alegre, Brazil; ^3^Department of Statistics, Institute of Exact Sciences (ICEx), Universidade Federal de Minas Gerais, Belo Horizonte, Brazil; ^4^Hospital das Clínicas da Faculdade de Medicina de Botucatu, Botucatu, Brazil; ^5^Hospital Evangélico de Belo Horizonte, Belo Horizonte, Brazil; ^6^Computer Science Department, Instituto de Ciências Exatas, Universidade Federal de Minas Gerais, Belo Horizonte, Brazil; ^7^Hospital Universitário de Santa Maria, Santa Maria, Brazil; ^8^Hospitais da Rede Mater Dei, Belo Horizonte, Brazil; ^9^Hospital de Clínicas de Porto Alegre, Porto Alegre, Brazil; ^10^Hospital Moinhos de Vento, Porto Alegre, Brazil; ^11^Hospital Santa Rosália, Teófilo Otoni, Brazil; ^12^Universidade Federal dos Vales do Jequitinhonha e Mucuri, Teófilo Otoni, Brazil; ^13^Hospital Márcio Cunha, Ipatinga, Brazil; ^14^Pontifícia Universidade Católica de Minas Gerais, Betim, Brazil; ^15^Hospital Santo Antônio, Curvelo, Brazil; ^16^Hospital SOS Cárdio, Florianópolis, Brazil; ^17^Hospital Universitário Ciências Médicas, Belo Horizonte, Brazil; ^18^Hospital São Lucas PUCRS, Porto Alegre, Brazil; ^19^Hospital Mãe de Deus, Porto Alegre, Brazil; ^20^Hospital Universitário de Canoas, Canoas, Brazil; ^21^Hospital Nossa Senhora da Conceição, Porto Alegre, Brazil; ^22^Hospital Bruno Born, Lajeado, Brazil; ^23^Hospital Santa Cruz, Santa Cruz do Sul, Brazil; ^24^Hospital Metropolitano Doutor Célio de Castro, Belo Horizonte, Brazil; ^25^Department of Internal Medicine, Medical School and Telehealth Center, University Hospital, Universidade Federal de Minas Gerais, Belo Horizonte, Brazil

**Keywords:** COVID-19, kidney transplantation, chronic kidney disease, dialysis, clinical characteristics, outcomes

## Abstract

Patients with chronic kidney disease (CKD), especially those on dialysis or who have received a kidney transplant (KT), are considered more vulnerable to severe COVID-19. This susceptibility is attributed to advanced age, a higher frequency of comorbidities, and the chronic immunosuppressed state, which may exacerbate their susceptibility to severe outcomes. Therefore, our study aimed to compare the clinical characteristics and outcomes of COVID-19 in KT patients with those on chronic dialysis and non-CKD patients in a propensity score-matched cohort study. This multicentric retrospective cohort included adult COVID-19 laboratory-confirmed patients admitted from March/2020 to July/2022, from 43 Brazilian hospitals. The primary outcome was in-hospital mortality. Propensity score analysis matched KT recipients with controls - patients on chronic dialysis and those without CKD (within 0.25 standard deviations of the logit of the propensity score) - according to age, sex, number of comorbidities, and admission year. This study included 555 patients: 163 KT, 146 on chronic dialysis, and 249 non-CKD patients (median age 57 years, 55.2% women). With regards to clinical outcomes, chronic dialysis patients had a higher prevalence of acute heart failure, compared to KT recipients, furthermore, both groups presented high in-hospital mortality, 34.0 and 28.1%, for KT and chronic dialysis patients, respectively. When comparing KT and non-CKD patients, the first group had a higher incidence of in-hospital dialysis (26.4% *vs*. 8.8%, *p* < 0.001), septic shock (24.1% *vs*. 12.0%, *p* = 0.002), and mortality (32.5% *vs*. 23.3%, *p* = 0.039), in addition to longer time spent in the intensive care unit (ICU). In this study, chronic dialysis patients presented a higher prevalence of acute heart failure, compared to KT recipients, whereas KT patients had a higher frequency of complications than those without CKD, including septic shock, dialysis during hospitalization, and in-hospital mortality as well as longer time spent in the ICU.

## Introduction

Since coronavirus disease 2019 (COVID-19) became a global pandemic, concerns have arisen regarding the risks of COVID-19 in chronic kidney disease (CKD) patients, especially kidney transplant (KT) and chronic dialysis patients (1). Previous studies have shown these patients are often more vulnerable to severe disease and mortality. This susceptibility is attributed to advanced age and higher frequency of comorbidities such as hypertension, diabetes, obesity, and cardiovascular diseases, all recognized as significant risk factors associated with worse COVID-19 prognosis. Furthermore, the chronic immunosuppressed state in these patients may exacerbate their susceptibility to severe outcomes associated with the virus (2–5).

However, evidence on the impact of immunosuppression on COVID-19 prognosis, as well as the disparities in outcomes among KT recipients and chronic dialysis patients remains a subject of contention in current research (6–8). Furthermore, during the COVID-19 pandemic, there was a reduction in morbidity and mortality rates related to improvements in the management, the emergence of variants, and especially vaccination of patients (9). The majority of studies focusing on KT recipients evaluated patients from the initial stages of the pandemic only, when therapeutic options and vaccination accessibility were still limited (10, 11).

In this context, it is imperative to evaluate the impact of the COVID-19 pandemic on KT recipients. There are still few studies on the impacts of COVID-19 on this population after 2020, especially in Latin America. Therefore, this study aimed to compare the clinical characteristics and outcomes of COVID-19 patients who received KT with those on chronic dialysis and patients non-CKD, in a propensity score-matched cohort study.

## Methods

### Study design

This retrospective cohort study included data from two cohorts. The “Brazilian COVID-19 Registry” multicentric cohort was developed in 41 Brazilian public and private hospitals, in 18 cities from six Brazilian states (Bahia, Minas Gerais, Pernambuco, Santa Caterina, São Paulo, Rio Grande do Sul) (12). The “COVID-19 in dialytic patients (VIDA) Study” was conducted in a private hospital in Belo Horizonte (Minas Gerais state) (13). The study adheres to the Strengthening the Reporting of Observational Studies in Epidemiology guidelines (STROBE) (14).

### Study participants

Consecutive adult (≥18 years old) patients with symptomatic and laboratory-confirmed COVID-19 (4, 15), in accordance with the World Health Organization guidance admitted to one of the participating hospitals between March 2020 to July 2022 were enrolled.

Patients who lost the kidney graft before contracting COVID-19, those who were transferred to other hospitals and whose outcomes are unknown, as well as those who manifested COVID-19 while hospitalized for other conditions, were excluded in this analysis. Furthermore, patients who underwent any type of transplant were not included in the control groups.

### Data collection

Trained researchers collected patient data from the hospital’s electronic records using Research Data Capture (REDCap) (16, 17), hosted at the Telehealth Center, University Hospital, *Universidade Federal de Minas Gerais* (17). Clinical variables including demographic and clinical characteristics, laboratory findings, therapeutic interventions, and outcomes were gathered, as described in details previously (18). Periodic audits were carried out to ensure data quality by detecting inconsistencies in values and missing information.

### Study groups

Kidney transplant patients were those with a previous history of KT, with no history of graft failure. Dialysis patients were those in chronic dialysis before COVID-19. Non-CKD patients were those with no evidence of CKD, per register in patients charts.

### Outcomes

The primary outcome was in-hospital mortality. Secondary outcomes included in-hospital dialysis, acute heart failure, hemorrhagic or thromboembolic events, septic shock, nosocomial infection, admission to the intensive care unit (ICU), time spent in the ICU, invasive mechanical ventilation IMV, and hospital length of stay, as previously defined (18).

### Statistical analysis

A propensity score model was estimated by logistic regression to adjust potential confounding variables and match: (i) KT recipients to chronic dialysis patients previous to COVID-19 admission, and (ii) KT recipients to non-CKD patients. The models included age, sex, number of comorbidities (hypertension, diabetes mellitus, obesity, coronary artery disease, heart failure, atrial fibrillation or flutter, cirrhosis, chronic obstructive pulmonary disease, cancer, and previous stroke), and admission year. Controls were searched to find those with the closest propensity score from the studies groups (within 0.25 standard deviations of the logit of the propensity score, on a scale from 0–1.00), using the MatchIt package in R software. Chronic dialysis controls were selected from both cohorts, but non-CKD controls were selected from the “Brazilian COVID-19 Registry” only, as “VIDA” Study did not include patients without CKD.

For the descriptive analysis, demographic, clinical characteristics and outcomes were represented by frequency distribution, using median and interquartile range for continuous variables, as they did not present normal distribution, and numbers and percentages for counts. Kidney transplant recipients were compared to matched controls (chronic dialysis patients previous to COVID-19 admission or non-CKD patients) using Fisher exact or Chi-square tests for categorical variables, and Wilcoxon or t-tests for continuous variables. In the latter case, the Kolmogorov–Smirnov test was applied to verify data normality. Significance level was established in a two-tailed *p*-value ≤0.05. All analyses were performed using R software (version 4.0.2).

### Ethics

Study protocols were approved by both the National Commission for Research Ethics (CAAE 30350820.5.1001.0008) and the *Associação Evangélica Beneficente de Minas Gerais* Research Ethics Committee (CAAE 31017120.9.0000.8787). Due to the pandemic situation and access to unidentified data, individual informed consent was waived.

## Results

**Figure 1:**
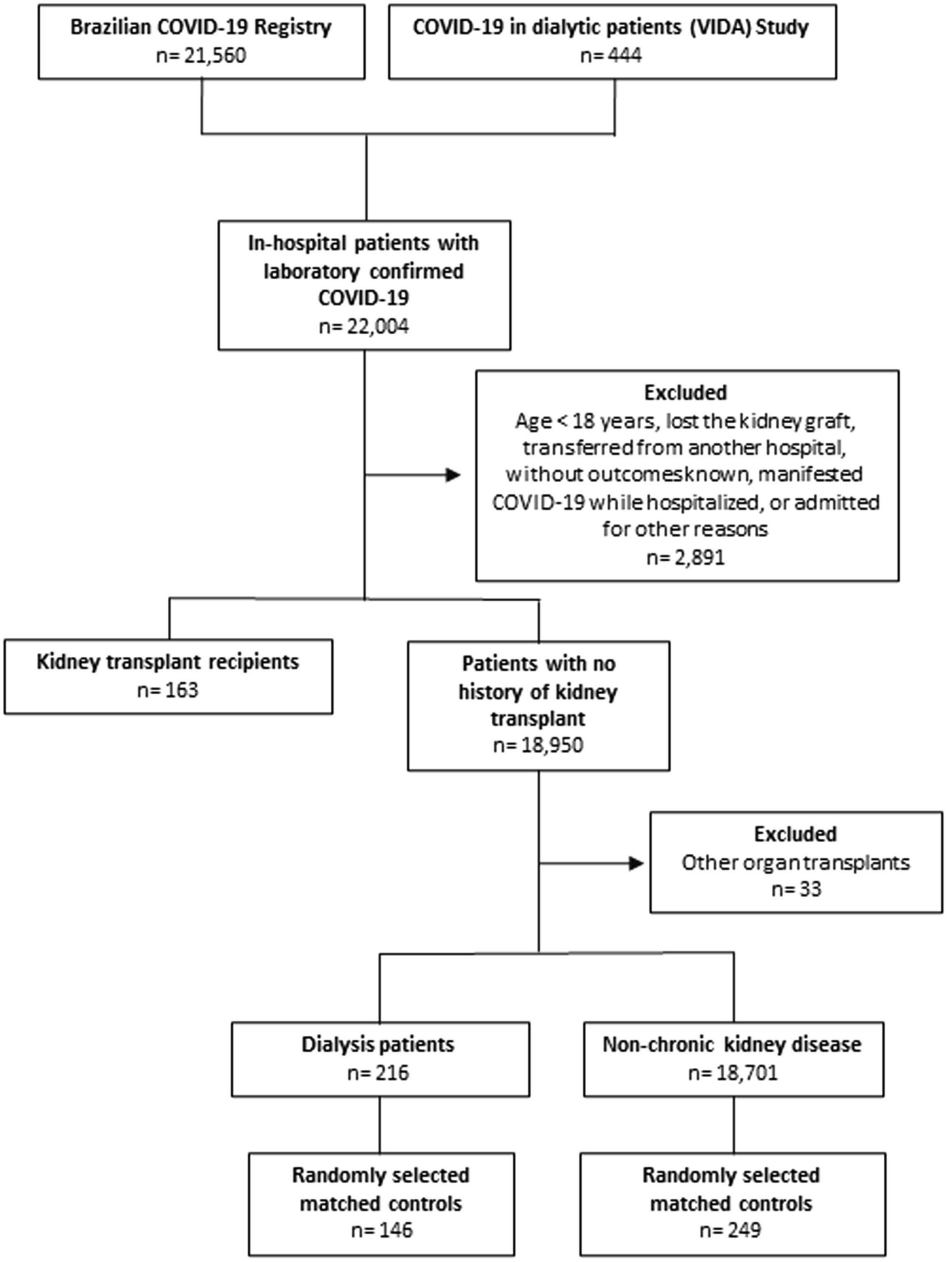
Flowchart of the patients included in the study.

Of the 22,004 patients admitted with a diagnosis of COVID-19, 163 were KT recipients (0.74%), 146 were selected as matched chronic dialysis controls, and 249 were selected as matched controls without CKD, as illustrated in Figure 1. The median time since transplant was 5.5 (IQR 3.0–9.3) years, ranging from 4 months to 28.0 years. In the comparison KT recipients *vs*. chronic dialysis controls, 144 patients could be matched, while in the comparison KT recipients *vs*. controls without CKD, all 163 were matched. Details on immunosuppressive therapy are shown in Supplementary Table S1. Out of the 152 patients from whom we obtained data on immunosuppressive therapy, 57.6% had their therapy suspended during hospitalization. In most cases, the decision to suspend therapy was based on the severity of the symptoms.

### Kidney transplant recipients *vs*. chronic dialysis patients

Chronic dialysis patients had a higher prevalence of underlying chronic obstructive pulmonary disease (COPD; 7.5% *vs*. 1.4%, *p* = 0.011) and rheumatological disease (4.8% *vs*. 0.0%, *p* = 0.015) than KT recipients. Diarrhea, hyporexia, and myalgia were more prevalent symptoms in KT recipients than chronic dialysis patients. Regarding the other demographic and clinical characteristics and other self-reported symptoms, there was no other significant statistical difference (Table 1; Supplementary Table S1). Chronic dialysis patients presented lower levels of hemoglobin, as well as higher levels of creatinine, blood urea, D-dimer, ferritin, C-reactive protein, leukocytes, and neutrophils. They also had a slightly higher activated partial thromboplastin time (aPTT), international normalized ratio (INR), sodium, sodium bicarbonate and pCO_2_ (Supplementary Table S2). Kidney transplant recipients had a higher frequency of corticosteroid usage compared to patients on dialysis (88.0% *vs*. 69.2%, *p* < 0.001), but there were no differences regarding other medications during hospitalization (Supplementary Table S3).

**Table 1:** Demographic data, clinical characteristics, and lifestyle habits of kidney transplant recipients *vs*. chronic dialysis patients, both infected with COVID-19.

Characteristics	Kidney transplant recipients^1^ (*n* = 144)^*^	Chronic dialysis patients^1^ (*n* = 146)	*p*-value^2^
Age (years)	5 (49, 65)	58 (49, 66)	0.669
Women	82 (56.9%)	83 (56.8%)	0.987
Year			0.791
2020	60 (41.7%)	63 (43.2%)	
2021	58 (40.3%)	61 (41.8%)	
2022	26 (18.1%)	22 (15.1%)	
Cardiovascular diseases			
Hypertension	110 (76.4%)	104 (71.2%)	0.318
Heart failure	6 (4.1%)	9 (6.0%)	0.434
Coronary artery disease	12 (8.8%)	16 (9.4%)	0.449
Ischemic stroke	4 (2.8%)	4 (2.7%)	>0.999
Atrial fibrillation or flutter	2 (1.4%)	6 (0.1%)	0.282
Respiratory diseases			
Asthma	3 (2.1%)	2 (1.4%)	0.683
COPD	2 (1.4%)	11 (7.5%)	0.011
Metabolic diseases			
Diabetes mellitus	75 (52.1%)	76 (52.1%)	0.996
Obesity	26 (18.1%)	15 (10.3%)	0.057
Other conditions			
Psychiatric disease	5 (4.1%)	5 (3.4%)	>0.999
Active cancer	4 (2.8%)	6 (4.1%)	0.750
Rheumatological disease	0 (0.0%)	7 (4.8%)	0.015
Cirrhosis	1 (0.7%)	0 (0.0%)	0.497
Number of comorbidities			0.707
0	14 (9.7%)	19 (13.0%)	
1	51 (35.4%)	41 (28.1%)	
2	53 (36.8%)	57 (39.0%)	
3	21 (14.6%)	23 (15.8%)	
4 or more	5 (3.5%)	6 (4.1%)	
Clinical characteristics at presentation			
GCS < 15	3 (2.5%)	11 (7.5%)	0.063
IMV	2 (1.8%)	67 (4.6%)	0.293
SpO_2_/FiO_2_	447.6 (407.1, 457.1)	428.6 (339.3, 452.4)	0.004
Lifestyle habits			
Alcohol use disorder	4 (3.3%)	8 (5.%)	0.386
Smoking	2 (1.4%)	5 (3.4%)	0.447
Vaccination			0.138
No	67 (46.5%)	67 (45.9%)	
Yes	23 (16.0%)	13 (8.9%)	
No information	54 (37.5%)	66 (45.2%)	
Which vaccine?	(*n* = 12)	(*n* = 6)	0.281
Astrazeneca	4 (33.3%)	1 (16.7%)	
Coronavac	5 (41.7%)	5 (83.3%)	
Pfizer	3 (25.0%)	0 (0.0%)	
Doses of the vaccine	(*n* = 23)	(*n* = 13)	0.764
1	4 (17.4%)	2 (15.4%)	
2	9 (39.1%)	3 (23.1%)	
3	6 (26.1%)	5 (38.5%)	
4	4 (17.4%)	3 (23.1%)	

^1^n (%); Median (IQR). ^2^Pearson’s Chi-squared test; Wilcoxon rank sum test; Fisher’s exact test. COPD, chronic obstructive pulmonary disease; GCS, Glasgow coma scale; FiO_2_, fraction of inspired oxygen; IMV, invasive mechanical ventilation; SpO_2_, peripheral oxygen saturation. *Although 163 patients were kidney transplant recipients, only 144 could be matched by age, sex, number of comorbidities, and admission year.

With regards to clinical outcomes, chronic dialysis patients had a higher prevalence of acute heart failure compared to KT recipients, while there was no difference between KT and chronic dialysis patients in the duration of hospital stay, ICU admission, time spent in the ICU, IMV, septic shock, nosocomial infection, thrombosis, and in-hospital death (Table 2).

**Table 2:** Clinical outcomes of kidney transplant recipients *vs*. chronic dialysis patients, both infected with COVID-19.

Characteristics	Kidney transplant recipients^1^ (*n* = 144)^*^	Chronic dialysis patients^1^ (*n* = 146)	*p*-value^2^
In-hospital stay (days)	11.0 (7.0, 18.0)	11.0 (5.2, 19.0)	0.847
ICU admission	65 (45.5%)	67 (45.9%)	0.941
Time spent in the ICU	10.0 (6.0, 18.0)	12.0 (4.0, 18.5)	0.612
IMV	52 (36.1%)	50 (34.7%)	0.805
Septic shock	31 (25.4%)	26 (17.8%)	0.130
Nosocomial infection	14 (9.7%)	23 (15.8%)	0.124
Acute heart failure	1 (0.8%)	9 (6.2%)	0.024
Thromboembolic events	2 (1.6%)	1 (0.7%)	0.593
Dialysis	36 (25.7%)	146 (100.0%)	NA
In-hospital death	49 (34.0%)	41 (28.1%)	0.274

^1^*n* (%); Median (IQR). ^2^Pearson’s Chi-squared test; Wilcoxon rank sum test; Fisher’s exact test. ICU, intensive care unit; IMV, invasive mechanical ventilation; NA, not applicable, as the control group comprehended patients on chronic dialysis. ^*^Although 163 patients were kidney transplant recipients, only 144 could be matched by age, sex, number of comorbidities, and admission year.

### Kidney transplant *vs*. non-chronic kidney disease patients

When comparing KT recipients to those without CKD, the KT group had a higher prevalence of underlying hypertension (75.5% *vs*. 43.4%, *p* < 0.001), and diabetes mellitus (DM) (45.6% *vs*. 23.9%, *p* = 0.015). Furthermore, the KT recipients had a slightly lower prevalence of, ischemic stroke (2.5% *vs*. 2.8%, *p* = 0.087), compared to the non-CKD patients. Demographic data, clinical characteristics, and lifestyle habits are shown in Table 3. When comparing laboratory findings at hospital admission, KT patients presented slightly lower levels of hemoglobin, platelets, INR, sodium, alanine aminotransferase, pCO_2_; lower white blood cells, neutrophils and lymphocytes count, and lower sodium bicarbonate; as well as higher levels of creatinine and blood urea (Supplementary Table S4), when compared to non-CKD patients. With regards to the frequency of self-reported symptoms at hospital admission, headache and dyspnea were more prevalent in those without CKD than the KT recipients, while the latter group had a higher prevalence of diarrhea and hyporexia (Supplementary Table S3). Kidney transplant recipients had a higher frequency of corticosteroid usage (89.5% *vs*. 79.1%, *p* = 0.007) and lower frequency of anticoagulant use (80.1% *vs*. 84.1%, *p* = 0.039) than patients without CKD (Supplementary Table S5).

**Table 3:** Demographic data, clinical characteristics, and lifestyle habits of kidney transplant recipients *vs*. non-chronic kidney disease patients, both infected with COVID-19.

Characteristics	Kidney transplant recipients^1^ (*n* = 163)	Non-chronic kidney disease patients^1^ (*n* = 249)	*p*-value^2^
Age (years)	56 (46.0, 64.5)	57 (41, 69)	0.776
Women	93 (57.1%)	132 (53.0%)	0.042
Admission year			0.024
2020	60 (36.8%)	108 (43.4%)	
2021	62 (38.0%)	106 (45.5%)	
2022	41 (25.2%)	36 (14.5%)	
Cardiovascular diseases			
Hypertension	123 (75.5%)	108 (43.4%)	<0.001
Heart failure	4 (2.5%)	12 (4.8%)	0.224
Coronary artery disease	13 (8.0%)	10 (4.0%)	0.087
Ischemic stroke	4 (2.5%)	7 (2.8%)	0.034
Atrial fibrillation or flutter	2 (1.2%)	5 (2.0%)	0.708
Respiratory diseases			
Asthma	3 (1.8%)	11 (4.4%)	0.158
COPD	2 (1.2%)	6 (2.4%)	0.487
Metabolic diseases			
Diabetes mellitus	76 (46.6%)	61 (24.5%)	<0.001
Obesity	27 (16.6%)	44 (17.7%)	0.771
Other conditions			
Psychiatric disease	6 (4.3%)	16 (6.4%)	0.372
Active cancer	4 (2.5%)	15 (6.0%)	0.091
Rheumatological disease	0 (0.0%)	5 (2.0%)	0.162
Cirrhosis	1 (0.6%)	1 (0.4%)	>0.999
Number of comorbidities			<0.001
0	19 (11.7%)	101 (40.6%)	
1	63 (38.7%)	68 (27.3%)	
2	55 (33.7%)	497 (19.7%)	
3	21 (12.9%)	21 (8.4%)	
4 or more	5 (3.1%)	10 (4.0%)	
Clinical characteristics at presentation			
GCS < 15	3 (2.1%)	14 (5.6%)	0.014
IMV	57 (35.0%)	80 (32.4%)	0.588
SpO_2_/FiO_2_	447.6 (414.3, 457.1)	346.4 (296.9, 438.1)	<0.001
Lifestyle habits			
Alcohol use disorder	5 (3.5%)	8 (3.2%)	0.860
Smoking	2 (1.2%)	8 (3.2%)	0.327
Vaccination			0.031
No	68 (41.7%)	133 (53.4%)	
Yes	31 (19.0%)	29 (11.6%)	
No information	64 (39.3%)	87 (34.9%)	
Which vaccine?	(*n* = 17)	(*n* = 11)	0.020
Astrazeneca	7 (41.2%)	4 (36.4%)	
Coronavac	5 (29.4%)	5 (45.5%)	
Janssen	0 (0.0%)	1 (9.1%)	
Pfizer	5 (29.4%)	1 (9.1%)	
Doses of the vaccine	(*n* = 31)	(*n* = 29)	0.132
1	4 (12.9%)	5 (17.2%)	
2	10 (32.3%)	14 (48.3%)	
3	12 (38.7%)	6 (20.7%)	
4	5 (16.1%)	4 (13.8%)	

^1^*n* (%); Median (IQR). ^2^Pearson’s Chi-squared test; Wilcoxon rank sum test; Fisher’s exact test. COPD, Chronic obstructive pulmonary disease; GCS, Glasgow coma scale; FiO_2_, fraction of inspired oxygen; IMV, invasive mechanical ventilation; SpO_2_, peripheral oxygen saturation. *Matched by age, sex, number of comorbidities, and admission year.

With regards to the outcomes, KT patients stayed longer in the ICU (10.0 *vs*. 7.0, *p* = 0.047) and had a longer hospital stay (11.0 *vs*. 8.0, *p* = 0.005). They also required a higher frequency of dialysis during hospitalization (26.4% *vs*. 8.8%, *p* < 0.001), and had a higher incidence of septic shock (24.1% *vs*. 12.0%, *p* = 0.002) and in-hospital death (32.5% *vs*. 23.3%, *p* = 0.039), than those without CKD (Table 4).

**Table 4:** Clinical outcomes of kidney transplant recipients *vs*. non-chronic kidney disease patients, both infected with COVID-19.

Characteristics	Kidney transplant recipients^1^ (*n* = 163)	Non-chronic kidney disease patients^1^ (*n* = 249)	*p*-value^2^
Duration of hospital stay	11.0 (7.0, 18.0)	8.0 (5.0, 15.0)	0.005
ICU admission	71 (43.8%)	120 (48.2%)	0.386
Time spent in the ICU	10.0 (6.0, 20.5)	7.0 (4.0, 17.0)	0.047
IMV	57 (35.0%)	80 (32.4%)	0.329
Septic shock	34 (24.1%)	30 (12.0%)	0.002
Nosocomial infection	15 (9.2%)	29 (11.6%)	0.432
Acute heart failure	1 (0.7%)	6 (2.4%)	0.430
Thromboembolic events	2 (1.4%)	11 (4.7%)	0.091
Dialysis	42 (26.4%)	22 (8.8%)	<0.001
In-hospital death	53 (32.5%)	58 (23.3%)	0.039

^1^*n* (%); Median (IQR). ^2^Pearson’s Chi-squared test; Wilcoxon rank sum test; Fisher’s exact test. ICU, intensive care unit; IMV, invasive mechanical ventilation. *Matched by age, sex, number of comorbidities, and admission year.

## Discussion

In this large Brazilian cohort, KT patients represented approximately 0.7% of the in-hospital COVID-19 patients. The matched analysis showed a similar incidence of severe outcomes in KT and dialysis patients, except for a higher incidence of acute heart failure in chronic dialysis patients. In-hospital mortality was high in both groups, 34.0 and 28.1%, respectively. Furthermore, KT patients had a higher incidence of in-hospital dialysis (26.4% *vs*. 8.8%, *p* < 0.001), septic shock (24.1% *vs*. 12.0%, *p* = 0.002), and in-hospital mortality (32.5% *vs*. 23.3%, *p* = 0.039), than those without CKD, as well as a longer time spent in the ICU (10.0 *vs*. 7.0, *p* = 0.047).

Kidney transplant recipients are known to be highly susceptible to infections and with increased risk of poorer prognosis, attributed not only to underlying comorbidities but also to the chronic use of immunosuppressive drugs (5). Brazil, with the world’s largest public organ transplant program, was significantly affected by the pandemic (19).

However, the majority of studies focusing on KT recipients were conducted in high-income nations, primarily during the early phases of the pandemic when treatment options and vaccinations were still limited. This raises concerns about the immunogenicity of vaccines in KT recipients, with unclear efficacy and effectiveness results in this population (20). Despite improvements in therapeutic management and immunization efforts, COVID-19 continues to pose a global threat, with KT recipients experiencing higher mortality rates compared to the general population (20, 21). Therefore, this multicenter cohort is of paramount importance in understanding the impact of COVID-19 on KT patients in Latin America, for having included patients after the first pandemic wave in Brazil and for having used propensity score matching in the analysis, controlling for potential confounders.

In the present study, mortality in KT and chronic dialysis was high, 34.0 and 28.1%, respectively, in contrast to 23.3% in non-CKD patients. These findings are in line with existing literature but surpass previously reported rates. For instance, a recent analysis involving data from the UK Kidney Association compared to age-matched general population from England observed COVID-19 mortality rates of 20 to 28% among KT recipients, significantly higher than the 1 to 5% observed in the general population (21). A meta-analysis encompassing 74 studies and 5,559 KT patients with COVID-19 from March 2020 to January 2021 reported an overall mortality rate of 23% (95% CI 21–27%), irrespective of gender, age, or comorbidities. Additionally, the risk of acute kidney injury (AKI) was 50% (95% CI 44–56%), a condition known to exacerbate renal outcomes and is an independent risk factor for mortality (22). Another study, an umbrella review of meta-analyses and systematic reviews, including patients from January 2020 to June 2022, observed a pooled mortality of 18% in KT recipients (23).

In our study, KT recipients also had a higher risk of complications from COVID-19, including dialysis during hospitalization, septic shock, and in-hospital mortality than those without CKD, as well as longer time spent in the ICU. The higher mortality and higher incidence of other complications have been attributed to the older age and the higher incidence of comorbidities in CKD patients (20, 24). In the present analysis, we have extended these findings, as we have shown that even after matching by age, sex, number of comorbidities, and admission year, patients with non-CKD had a better prognosis.

Overall, KT recipients usually have a more favorable clinical profile than chronic dialysis patients. In the present analysis, a robust method with a propensity score matching analysis was used to adjust for possible confounders, and there were no significant differences between KT and chronic dialysis in in-hospital mortality and the secondary outcomes, except for acute heart failure. Our results corroborate Hilbrand’s study, based on the ERACODA database, a large European data set containing detailed individual patient data, facilitating well-powered analyses of risk factors for mortality in chronic dialysis and transplant patients with COVID-19. Its results showed that mortality in KT and chronic dialysis patients with COVID-19 was high (21.3 and 25.0%, respectively) and primarily associated with advanced age and frailty, while hypertension, diabetes mellitus, coronary artery disease, heart failure, and chronic lung disease did not emerge as independent risk factors in its analysis. After adjusting for age, sex, and frailty, the authors observed no significant difference in in-hospital mortality between KT and chronic dialysis patients (2).

A Brazilian cohort used data from 65 dialysis units and 43 transplant centers and observed that chronic dialysis patients had higher 30-day mortality (6% higher per day) than matched KT recipients. However, patients were included in the first COVID-19 wave only, and there is a high risk of sampling bias: the sample of transplanted patients was greater than the sample of patients on chronic dialysis, which is the opposite of what would be expected. Actually, the number of chronic dialysis patients was quite low, considering the number of dialysis units included (25). Therefore, there might be a problem with the representativeness of the chronic dialysis patients included, i.e., researchers might have included more frequently those with other risk factors for severe COVID-19 or, retrospectively, those who required hospital admission. Additionally, the number of variables used for matching (age, gender, ethnicity, body mass index, comorbidities, and geographic region of the transplant/dialysis site) might have been excessive, which might have impaired the observation of differences in clinical characteristics between groups.

The higher frequency of acute heart failure in chronic dialysis patients can prompt discussion regarding specific conditions related to these patients. Cardiorenal syndrome refers to a well-established pathophysiological condition involving both the kidneys and the heart. In this syndrome, significant dysfunction – acute or chronic – in one of these organs can precipitate corresponding dysfunction in the other (26). Type IV cardiorenal syndrome describes the condition in which chronic kidney disease can contribute to the deterioration of cardiac function, significantly elevating the risk of acute cardiovascular events, including acute heart failure (26). Furthermore, chronic dialysis patients are susceptible to uremia-related cardiomyopathy (27). Recent research demonstrates an improvement in cardiomyopathy following kidney transplantation, emphasizing the crucial role of improved renal function. We hypothesize that cardiorenal syndrome, combined with anuria in some patients and a higher frequency of hypervolemia, along with the observed lower levels of hemoglobin, predisposed them to cardiac decompensation (27).

Despite the growing literature on the prognosis of COVID-19 in KT patients, it remains unclear whether immunosuppressive treatment is an independent risk factor for a poor prognosis of COVID-19. In general, immunocompromised patients are a heterogeneous population, with significant variations in the severity of COVID-19 among individuals in this group. In this context, Center for Disease Control and Prevention (CDC) listed immunocompromised patients as high risk for severe disease from SARS-CoV-2 (28). There is evidence that certain classes of immunosuppressants, such as T cell–suppressing agents or T cell–depletion, and B cell–depleting agents, are associated with more severe COVID-19 (29, 30). Similarly, individuals who make autoantibodies to type I interferons have been shown to have a higher risk of severe COVID-19 (28). Another point of attention is the lower immunogenicity rates after SARS-CoV-2 vaccination in patients who are moderately or severely immunocompromised (28). However, all these topics are insights for future analyses.

With regards to patients on chronic dialysis, evidence shows that patients experience a pro-inflammatory state due to an increase in cytokines associated with an irreversible reduction in immune defense given to functional alterations in almost all populations of innate and acquired immune cells, and the premature aging of the immune system. Consequently, patients with end-stage renal disease are more susceptible to infections and unfavorable evolution, including COVID-19, which may explain the high mortality in this group of patients (29–31).

This study has some limitations that should be considered. It was a retrospective analysis, subject to the drawbacks of a patient records review. Thus, we were unable to obtain data on the impact of the cessation of immunosuppressive medication during hospitalization a challenge that was observed in clinical practice and also reported by previous studies (2). This is an important topic for future studies. It is important to assess whether it is effective for improving the COVID-19 course, reducing the incidence of COVID-19 complications; and whether it has an impact on the kidney graft function and in the risk of rejection. Furthermore, we did not assess data about the time spent on dialysis an interesting and complementary question for new analyses.

Conversely, this study has significant strengths. One of its main strengths is the fact that it is based on a database of real-life cases, encompassing comprehensive data from patients from 41 different hospitals, located in different regions of Brazil, which ensures the diversity of the population studied. The data were obtained employing a detailed medical record review, which results in a higher degree of detail than would the electronic abstraction of structured data elements. Additionally, the data was submitted to periodic auditing to ensure data quality, and the propensity score matched analysis addressed critical confounding factors.

We believe our findings can contribute significantly to the existing literature on several fronts. Primarily, our study encompasses data from Latin America, with a particular focus on Brazil, a nation profoundly affected by the COVID-19 pandemic. Despite the challenges posed by the pandemic, our study provides valuable data, including insights from COVID-19 KT recipients, which are still relatively scarce in the literature. Second, previous studies that investigated the association of the type of kidney replacement therapy (dialysis or transplant) with COVID-19 have controversial results. Some studies have indicated higher mortality rates in KT patients, while others have reported similar outcomes between the two groups. For instance, data from the European Renal Association COVID-19 Database, which included 496 KT recipients and 1,174 dialysis patients diagnosed with COVID-19 between February 1, 2020, and December 1, 2020, revealed a 78% higher risk of death in KT recipients compared to dialysis patients [hazard ratio 1.78 (95% CI 1.22–2.61)], after fully adjusting for confounding factors (32).

Furthermore, in the present analysis, we used a robust method with a propensity score matching analysis to adjust for possible confounders, and there were no significant differences between KT and chronic dialysis in in-hospital mortality, as well as the secondary outcomes except for acute heart failure. In this line, our results confirm and extend Hilbrand’s study, based on the ERACODA database, a large European dataset. After adjusting for age, sex, and frailty, the authors observed no significant difference in in-hospital mortality between KT and chronic dialysis patients (2). This study included patients from the first pandemic wave in Brazil only, and our study also included data from the second and third waves. Additionally, the present study also assessed other outcomes, including in-hospital dialysis, acute heart failure, hemorrhagic or thromboembolic events, septic shock, nosocomial infection, admission to the ICU, time spent in the ICU, IMV, and hospital length of stay.

## Conclusion

In our study, there were no differences in the clinical outcomes among KT and chronic dialysis patients, except for acute heart failure, whereas KT patients had a higher frequency of complications than those without CKD, including a higher incidence of septic shock, dialysis during hospitalization, in-hospital death, as well as longer time spent in the ICU.

## Data availability statement

The raw data supporting the conclusions of this article will be made available by the authors, without undue reservation.

## Ethics statement

The studies involving humans were approved by Comissão Nacional de Ética em Pesquisa, approval number CAAE 30350820.5.0000.0008 Associação Evangélica Beneficente de Minas Gerais Research Ethics Committee CAAE 31017120.9.0000.8787. The studies were conducted in accordance with the local legislation and institutional requirements. Written informed consent for participation was not required from the participants or the participants’ legal guardians/next of kin because due to the pandemic situation and access to unidentified data, individual informed consent was waived.

## Author contributions

PDP: Conceptualization, Methodology, Investigation, Formal analysis, Project administration, Supervision, Visualization, Writing – original draft, Writing – review & editing. VGJV: Conceptualization, Methodology, Investigation, Formal analysis, Project administration, Supervision, Visualization, Writing – original draft, Writing – review & editing. MCP: Methodology, Formal analysis, Software, Writing – review & editing. DP: Conceptualization, Methodology, Investigation, Formal analysis, Writing – review & editing. GALC: Methodology, Investigation, Formal analysis, Writing – review & editing. LPFC: Formal analysis, Methodology, Investigation, Writing – review & editing. BBMP: Methodology, Formal analysis, Software, Writing – review & editing. AVS: Methodology, Investigation, Formal analysis, Writing – review & editing. AGRG: Methodology, Investigation, Formal analysis, Writing – review & editing. BMC: Methodology, Investigation, Formal analysis, Writing – review & editing. CAP: Methodology, Investigation, Formal analysis, Writing – review & editing. CCRM: Methodology, Investigation, Formal analysis, Writing – review & editing. DAL: Methodology, Investigation, Formal analysis, Writing – review & editing. FCS: Methodology, Investigation, Formal analysis, Writing – review & editing. FB: Methodology, Investigation, Formal analysis, Writing – review & editing. GGV: Methodology, Investigation, Formal analysis, Writing – review & editing. HRV: Methodology, Investigation, Formal analysis, Writing – review & editing. JMC: Methodology, Investigation, Formal analysis, Writing – review & editing. KBR: Methodology, Investigation, Formal analysis, Writing – review & editing. LK: Methodology, Investigation, Formal analysis, Writing – review & editing. LCC: Methodology, Investigation, Formal analysis, Writing – review & editing. MC: Methodology, Investigation, Formal analysis, Writing – review & editing. MSM: Conceptualization, Data curation, Funding acquisition, Methodology, Investigation, Formal analysis, Project administration, Resources, Supervision, Visualization, Writing – original draft, Writing – review & editing.
