# Supplementary material for: Clinical characteristics and outcomes in COVID-19 in kidney transplant recipients: a propensity score matched cohort study
Source: Front Med (Lausanne). 2024 Apr 15;11:1350657. doi: 10.3389/fmed.2024.1350657 (PMC11056524; doi:10.3389/fmed.2024.1350657)
Supplement: Supplementary file 1 [file Table_1.docx]

**SUPPLEMENTARY MATERIAL**

| **Table S1.** Transplant duration and immunosuppressive therapy in kidney transplant patients. | | |
| --- | --- | --- |
| **Variables** | **n (%) or median (IQR)** | **Non-missing cases** |
| ***Time since transplant*** | 5.5 (3.0-9.3) | 152/163 (93.3%) |
| ***Previous history of lymphocyte-depleting therapy*** | 33 (34.4%) | 96/163 (58.9%) |
| ***Immunosuppressive drugs before hospitalization*** |  | 152/163 (93.3%) |
| Tacrolimus | 112 (73.7%) |  |
| Sirolimus | 30 (19.9%) |  |
| Everolimus | 8 (5.3%) |  |
| Mycophenolate | 99 (65.1%) |  |
| Azathioprine | 13 (8.6%) |  |
| Cyclosporin | 14 (9.3%) |  |
| Prednisone | 104 (68.9%) |  |
| Deflazacort | 2 (1.3%) |  |
| ***Immunosuppressive drugs at hospital discharge*** |  | 92/110^b^ (83.6%) |
| Tacrolimus | 70 (63.6%) |  |
| Sirolimus | 17 (15.5%) |  |
| Everolimus | 6 (5.5%) |  |
| Mycophenolate | 56 (50.9%) |  |
| Azathioprine | 10 (9.1%) |  |
| Cyclosporin | 4 (3.6%) |  |
| Prednisone | 59 (53.6%) |  |
| Deflazacort | 1 (0.9%) |  |
| Dexamethasone | 1 (0.9%) |  |
| ***Immunosuppressive therapy before hospitalization*** |  | 152/163 (93.3%) |
| Azathioprine, Cyclosporin | 3 (2.0%) |  |
| Cyclosporin | 1 (0.7%) |  |
| Cyclosporin, Mycophenolate | 8 (5.4%) |  |
| Cyclosporin, Sirolimus | 2 (1.4%) |  |
| Everolimus, Mycophenolate | 3 (2.0%) |  |
| Mycophenolate | 7 (4.7%) |  |
| Sirolimus | 4 (2.7%) |  |
| Sirolimus, Mycophenolate | 8 (5.4%) |  |
| Tacrolimus | 8 (5.4%) |  |
| Tacrolimus, Azathioprine | 10 (6.8%) |  |
| Tacrolimus, Everolimus | 5 (3.4%) |  |
| Tacrolimus, Mycophenolate | 73 (49.3%) |  |
| Tacrolimus, Sirolimus | 16 (10.8%) |  |
| ***Immunosuppressive regimen modified during hospitalization*** | 92 (64.7%) | 150/163 (92.0%) |
| Started mycophenolate | 2 (1.3%) |  |
| Changed from prednisone to dexamethasone | 49 (33.1%) |  |
| ***Immunosuppressive therapy temporary suspended during hospitalization*** | 83 (57.6%) | 144/163 (88.3%) |
| *Complete* | 35/151 (23.2%) |  |
| Tacrolimus only | 4/111^a^ (3.6%) |  |
| Sirolimus only | 3/30^a^ (10.0%) |  |
| Everolimus only | 1/8^a^ (12.5%) |  |
| Mycophenolate only | 30/98^a^ (30.6%) |  |
| Azathioprine only | 4/13^a^ (30.8%) |  |
| Cyclosporin only | 3/14^a^ (21.4%) |  |
| ***Immunosuppressive therapy at hospital discharge*** |  | 92/110^b^  (83.6%) |
| Azathioprine, Cyclosporin | 2 (1.8%) |  |
| Cyclosporin | 1 (0.9%) |  |
| Cyclosporin, Sirolimus | 1 (0.9%) |  |
| Everolimus | 1 (0.9%) |  |
| Everolimus, Mycophenolate | 1 (0.9%) |  |
| Mycophenolate | 5 (4.5%) |  |
| Sirolimus | 4 (3.6%) |  |
| Sirolimus, Mycophenolate | 6 (5.5%) |  |
| Tacrolimus | 8 (7.3%) |  |
| Tacrolimus, Azathioprine | 8 (7.3%) |  |
| Tacrolimus, Everolimus | 4 (3.6%) |  |
| Tacrolimus, Mycophenolate | 44 (40.0%) |  |
| Tacrolimus, Sirolimus | 6 (5.5%) |  |

^a^Number of patients who had temporary suspension of the drug, excluding those who had complete suspension of the immunosuppressive regimen/Number of patients using the drug alone or in combination. ^b^The denominator excludes deceived patients.
