# Supplementary material for: Clinical characteristics and outcomes in COVID-19 in kidney transplant recipients: a propensity score matched cohort study
Source: Front Med (Lausanne). 2024 Apr 15;11:1350657. doi: 10.3389/fmed.2024.1350657 (PMC11056524; doi:10.3389/fmed.2024.1350657)
Supplement: Supplementary file 2 [file Table_2.docx]

**SUPPLEMENTARY MATERIAL**

| **Table S2.** Symptoms and laboratory findings at hospital admission of kidney transplant recipients *vs.* chronic dialysis patients, both infected with COVID-19. | | | |
| --- | --- | --- | --- |
| **Characteristics** | **Kidney transplant recipients^1^ (n= 144)** | **Chronic dialysis patients^1^ (n= 146)** | **p-value^2^** |
| *Symptoms* |  |  |  |
| Adynamic | 29 (23.8%) | 37 (25.3%) | 0.776 |
| Ageusia | 7 (4.9%) | 4 (2.7%) | 0.344 |
| Anosmia | 10 (6.9%) | 5 (3.4%) | 0.176 |
| Arthralgia | 0 (0.0%) | 1 (0.7%) | >0.999 |
| Headache | 18 (12.5%) | 14 (9.6%) | 0.429 |
| Rhinorrhea | 16 (13.1%) | 14 (9.6%) | 0.362 |
| Diarrhea | 52(36.1%) | 21 (14.4%) | <0.001 |
| Dyspnea | 69 (47.9%) | 72 (49.3%) | 0.812 |
| Sore throat | 6 (4.2%) | 3 (2.1%) | 0.333 |
| Fever | 67 (46.5%) | 70 (47.9%) | 0.809 |
| Hyporexia | 25 (20.1%) | 17 (11.6%) | 0.047 |
| Neurological manifestations | 0 (0.0%) | 5 (3.4%) | 0.065 |
| Myalgia | 33 (27.0%) | 23 (15.8%) | 0.024 |
| Nausea/vomiting | 21 (14.6%) | 18 (12.3%) | 0.574 |
| Cough | 70 (48.6%) | 61 (41.8%) | 0.243 |
| Asymptomatic | 1 (0.8%) | 0 (0.0%) | 0.455 |
| CGS<15 | 3 (2.5%) | 11 (7.5%) | 0.063 |
| *Laboratory findings* |  |  |  |
| Hemoglobin (g/dL) | 12.4 (10.9,13.8) | 10.1 (8.4, 11.5) | <0.001 |
| Leucocytes (cels/mm^3^) | 5,260.0 (3,555.0, 7,685.0) | 6,300.0 (4,380.0, 9,140.0) | 0.005 |
| Neutrophils (cels/mm^3^) | 3,546.5 (2,605.8, 6,207.5) | 4,780.0 (3,085.0, 7,506.0) | 0.007 |
| Lymphocytes (cels/mm^3^) | 697.6 (474.2, 1,026.0) | 737.0 (494.0, 1,264.2) | 0.329 |
| Platelets (cels/mm^3^) | 170,000.0 (138,500.0, 226,000.0) | 176,000.0 (137,350.0, 238,000.0) | 0.626 |
| Bilirubin (mg/dL) | 0.5 (0.3, 0.6) | 0.4 (0.3, 0.6) | 0.886 |
| Creatinine (mg/dL) | 1.7 (1.3, 2.6) | 6.5 (3.8, 10.8) | <0.001 |
| D-dimer (ng/mL) | 442.0 (1.2, 930.0) | 1,101.8 (689.0, 2,589.0) | <0.001 |
| Feritin (ng/mL) | 265.4 (188.4, 642.4) | 962.5 (126.0, 2,000.0) | 0.467 |
| Lactate (mmol/L) | 1.5 (1.2, 2.0) | 1.4 (1.1, 2.0) | 0.359 |
| CRP (mg/L) | 72.0 (41.1, 159.8) | 132.1 (67.0, 184.8) | 0.001 |
| aPTT (seconds/control) | 1.0 (1.0, 1.2) | 1.1 (1.0, 1.3) | 0.050 |
| INR | 1.0 (1.0, 1.1) | 1.1 (1.1, 1.2) | 0.003 |
| Sodium (mmol/L) | 135.0 (131.0, 137.0) | 135.9 (133.6, 138.6) | 0.020 |
| AST (U/L) | 37.0 (23.0, 53.7) | 31.8 (23.0, 43.2) | 0.181 |
| ALT (U/L) | 26.0 (17.9, 36.7) | 17.0 (13.0, 25.0) | 0.003 |
| Urea (mg/dL) | 62.0 (41.8, 92.2) | 93.0 (66.8, 134.5) | <0.001 |
| pH | 7.4 (7.4, 7.4) | 7.4 (7.3, 7.5) | 0.033 |
| pCO_2_ | 31.6 (28.1, 34.1) | 35.0 (29.7, 40.0) | 0.001 |
| pO_2_ | 78.7 (67.7, 95.2) | 78.5 (59.8, 103.0) | 0.782 |
| HCO_3_^-^ | 19.0 (16.4, 21.0) | 22.0 (18.3, 26.0) | <0.001 |
| ^1^n (%); Median (IQR). ^2^Pearson's Chi-squared test; Wilcoxon rank sum test; Fisher's exact test. AST: Aspartate aminotransferase; ALT: Alanine aminotransferase; CGS: Glasgow Coma Scale; CRP: C reactive protein; HCO_3_^-^: Bicarbonate; INR: International normalized ratio; pCO_2_: Partial pressure of carbon dioxide; pH: Potential hydrogen; pO_2_: Partial pressure of oxygen. *Matched by age, sex, number of comorbidities, and admission year. | | | |
