# Supplementary material for: Clinical characteristics and outcomes in COVID-19 in kidney transplant recipients: a propensity score matched cohort study
Source: Front Med (Lausanne). 2024 Apr 15;11:1350657. doi: 10.3389/fmed.2024.1350657 (PMC11056524; doi:10.3389/fmed.2024.1350657)
Supplement: Supplementary file 3 [file Table_3.docx]

**SUPPLEMENTARY MATERIAL**

| **Table S3.** Therapy during hospitalization for kidney transplant recipients *vs.* chronic dialysis patients, both infected with COVID-19. | | | |
| --- | --- | --- | --- |
| **Characteristics** | **Kidney transplant recipients^1^ (n= 144)** | **Chronic dialysis patients^1^ (n= 146)** | **p-value^2^** |
| Anticoagulant | 100 (82.0%) | 123 (84.2%) | 0.613 |
| Oral or intravenous corticosteroids | 117 (88.0%) | 101 (69.2%) | <0.001 |
| Immunoglobulin | 0 (0.0%) | 0 (0.0%) | - |
| Convalescent plasma | 1 (0.8%) | 0 (0.0%) | 0.455 |
| Remdesivir | 0 (0.0%) | 0 (0.0%) | - |
| Sarilumab | 0 (0.0%) | 0 (0.0%) | - |
| Tocilizumab | 1 (0.8%) | 0 (0.0%) | 0.455 |
| ^1^n (%); Median (IQR). ^2^Pearson's Chi-squared test; Wilcoxon rank sum test; Fisher's exact test. *Matched by age, sex, number of comorbidities, and admission year. | | | |
