# Supplementary material for: Clinical characteristics and outcomes in COVID-19 in kidney transplant recipients: a propensity score matched cohort study
Source: Front Med (Lausanne). 2024 Apr 15;11:1350657. doi: 10.3389/fmed.2024.1350657 (PMC11056524; doi:10.3389/fmed.2024.1350657)
Supplement: Supplementary file 4 [file Table_4.docx]

**SUPPLEMENTARY MATERIAL**

| **Table S4.** Symptoms and laboratory findings at hospital admission of kidney transplant recipients *vs.* non-chronic kidney disease patients, both infected with COVID-19. | | | | | |
| --- | --- | --- | --- | --- | --- |
| **Characteristics** | **Kidney transplant recipients^1^ (n= 163)** | | **Non-chronic kidney disease patients^1^ (n= 249)** | | **p-value^2^** |
| *Symptoms* |  | |  | |  |
| Adynamic | 32 (22.7%) | | 41 (16.5%) | | 0.130 |
| Ageusia | 9 (5.5%) | | 21 (8.4%) | | 0.266 |
| Anosmia | 12 (7.4%) | | 26 (10.4%) | | 0.291 |
| Arthralgia | 0 (0.0%) | | 2 (0.8%) | | 0.537 |
| Headache | 20 (12.3%) | | 60 (24.1%) | | 0.003 |
| Rhinorrhea | 18 (12.8%) | | 29 (11.6%) | | 0.744 |
| Diarrhea | 63 (38.7%) | | 29 (11.6%) | | <0.001 |
| Dyspnea | 79 (48.5%) | | 171 (68.7) | | <0.001 |
| Sore throat | 11 (6.7%) | | 27 (4.7%) | | 0.160 |
| Fever | 75 (46.0%) | | 125 (50.2%) | | 0.406 |
| Hyporexia | 29 (20.6%) | | 26 (10.4%) | | 0.006 |
| Neurological manifestations | 0 (0.0%) | | 2 (0.8%) | | 0.537 |
| Myalgia | 40 (28.4%) | | 67 (26.2%) | | 0.756 |
| Nausea/vomiting | 27 (16.6%) | | 25 (10.0%) | | 0.051 |
| Cough | 82 (50.3%) | | 103 (41.2%) | | 0.074 |
| Asymptomatic | 1 (0.7%) | | 0 (0.0%) | | 0.362 |
| CGS<15 | 3 (2.1%) | | 14 (5.6%) | | 0.104 |
| *Laboratory findings* |  |  | |  | |
| Hemoglobin (g/dL) | 12.4 (10.8, 13.8) | | 13.1 (12.0, 14.6) | | <0.001 |
| Leucocytes (cels/mm^3^) | 5,270.0 (3,555.0, 7,740.0) | | 7,450.0 (5,250.8, 10,520.3) | | <0.001 |
| Neutrophils (cels/mm^3^) | 3,583.0 (2,613.5, 6,253.2) | | 5,600.0 (3,689.5, 8,266.8) | | <0.001 |
| Lymphocytes (cels/mm^3^) | 712.0 (474.2, 1,029.5) | | 968.0 (739.0, 1,399.0) | | <0.001 |
| Platelets (cels/mm^3^) | 170,000.0 (138,000.0, 223,000.0) | | 192,000.0 (148,000,0, 271,250.0) | | 0.017 |
| Bilirubin (mg/dL) | 0.4 (0.3, 0.6) | | 0.4 (0.3, 0.6) | | 0.911 |
| Creatinine (mg/dL) | 1.7 (1.3, 2.8) | | 0.9 (0.7, 1.1) | | <0.001 |
| D-dimer (ng/mL) | 502.0 (1.3, 927.5) | | 313.0 (1.2, 958.0) | | 0.342 |
| Feritin (ng/mL) | 265.4 (171.7, 858.6) | | 985.1 (303.4, 1,518.4) | | 0.342 |
| Lactate (mmol/L) | 1.5 (1.2, 2.0) | | 1.4 (1.0, 1.8) | | 0.135 |
| CRP (mg/L) | 72.0 (39.9, 154.6) | | 77.8 (45.4, 147.6) | | 0.439 |
| PTTa (seconds)/control | 1.0 (1.0, 1.2) | | 1.0 (0.9, 1.1) | | 0.247 |
| INR | 1.0 (1.0, 1.1) | | 1.1 (1.0, 1.2) | | 0.002 |
| Sodium (mmol/L) | 135.0 (131.2, 137.0) | | 137.0 (134.7, 139.0) | | <0.001 |
| AST (U/L) | 37.0 (23.0, 53.7) | | 38.6 (30.0, 55.0) | | 0.187 |
| ALT (U/L) | 26.0 (18.0, 37.5) | | 37.0 (24.9, 67.5) | | <0.001 |
| Urea (mg/dL) | 63.0 (42.2, 87.8) | | 34.0 (25.0, 48.8) | | <0.001 |
| pH | 7.4 (7.4, 7.4) | | 7.4 (7.4, 7.5) | | <0.001 |
| pCO_2_ | 31.5 (27.3, 34.3) | | 36.0 (32.2, 39.0) | | <0.001 |
| pO_2_ | 79.2 (67.7, 95.8) | | 75.8 (63.7, 101.2) | | 0.597 |
| HCO_3_^-^ | 18.9 (15.9, 21.2) | | 23.6 (21.8, 25.6) | | <0.001 |
| ^1^n (%); Median (IQR). ^2^Pearson's Chi-squared test; Wilcoxon rank sum test; Fisher's exact test. AST: Aspartate aminotransferase; ALT: Alanine aminotransferase; CGS: Glasgow Coma Scale; CRP: C reactive protein; HCO_3_^-^: Bicarbonate; INR: International normalized ratio; pCO_2_: Partial pressure of carbon dioxide; pH: Potential hydrogen; pO_2_: Partial pressure of oxygen. *Matched by age, sex, number of comorbidities, and admission year. | | | | | |
