# Supplementary material for: Clinical characteristics and outcomes in COVID-19 in kidney transplant recipients: a propensity score matched cohort study
Source: Front Med (Lausanne). 2024 Apr 15;11:1350657. doi: 10.3389/fmed.2024.1350657 (PMC11056524; doi:10.3389/fmed.2024.1350657)
Supplement: Supplementary file 5 [file Table_5.docx]

**SUPPLEMENTARY MATERIAL**

| **Table S5.** Therapy during hospitalization for kidney transplant recipients *vs.* non-chronic dialysis patients, both infected with COVID-19. | | | |
| --- | --- | --- | --- |
| **Characteristics** | **Kidney transplant recipients^1^ (n= 163)** | **Non-chronic kidney disease patients^1^ (n= 249)** | **p-value^2^** |
| Anticoagulant | 113 (80.1%) | 227 (84.1%) | 0.039 |
| Oral or intravenous corticosteroids | 136 (89.5%) | 196 (79.1%) | 0.007 |
| Immunoglobulin | 0 (0.0%) | 0 (0.0%) | - |
| Convalescent plasma | 1 (0.7%) | 1 (0.0%) | 0.362 |
| Remdesivir | 0 (0.0%) | 0 (0.0%) | - |
| Sarilumab | 0 (0.0%) | 0 (0.4%) | - |
| Tocilizumab | 1 (0.7%) | 25 (0.8%) | >0.999 |
| ^1^n (%); Median (IQR). ^2^Pearson's Chi-squared test; Wilcoxon rank sum test; Fisher's exact test. *Matched by age, sex, number of comorbidities, and admission year. | | | |
