# Supplementary material for: Clinical characteristics and outcomes in COVID-19 in kidney transplant recipients: a propensity score matched cohort study
Source: Front Med (Lausanne). 2024 Apr 15;11:1350657. doi: 10.3389/fmed.2024.1350657 (PMC11056524; doi:10.3389/fmed.2024.1350657)
Supplement: Supplementary file 6 [file Table_6.docx]

**SUPPLEMENTARY MATERIAL**

| **Table S6.** STROBE Statement - Checklist of items that should be included in reports of observational studies. | | | |
| --- | --- | --- | --- |
|  | **Item** | **Recommendation** | **Page** |
| **Title and abstract** | 1 | (a) Indicate the study’s design with a commonly used term in the title or the abstract | 1 |
|  |  | (b) Provide in the abstract an informative and balanced summary of what was done and what was found | 1-2 |
| **Introduction** | | | |
| Background/  Rationale | 2 | Explain the scientific background and rationale for the investigation being reported | 2 |
| Objectives | 3 | State specific objectives, including any prespecified hypotheses | 2 |
| **Methods** | | | |
| Study design | 4 | Present key elements of study design early in the paper | 2 |
| Setting | 5 | Describe the setting, locations, and relevant dates, including periods of recruitment, exposure, follow-up, and data collection | 2-3 |
| Participants | 6 | (a) Cohort study—Give the eligibility criteria, and the sources and methods of selection of participants. Describe methods of follow-up | 2-3 |
|  |  | (b) Cohort study—For matched studies, give matching criteria and number of exposed and unexposed | 2-3 |
| Variables | 7 | Clearly define all outcomes, exposures, predictors, potential confounders, and effect modifiers. Give diagnostic criteria, if applicable | 2-3 |
| Data sources/ measurement | 8 | For each variable of interest, give sources of data and details of methods of assessment (measurement). Describe comparability of assessment methods if there is more than one group | 2-3 |
| Bias | 9 | Describe any efforts to address potential sources of bias | 2-3 |
| Study size | 10 | Explain how the study size was arrived at | 2-3  Figure 1 |
| Quantitative variables | 11 | Explain how quantitative variables were handled in the analyses. If applicable, describe which groupings were chosen and why | 2-3 |
| Statistical methods | 12 | (a) Describe all statistical methods, including those used to control for confounding | 3 |
|  |  | (b) Describe any methods used to examine subgroups and interactions | 3 |
|  |  | (c) Explain how missing data were addressed | NA |
|  |  | (d) Cohort study—If applicable, explain how loss to follow-up was addressed | NA |
|  |  | (e) Describe any sensitivity analyses | NA |
| **Results** |  |  |  |
| Participants | 13 | (a) Report numbers of individuals at each stage of study—eg numbers potentially eligible, examined for eligibility, confirmed eligible, included in the study, completing follow-up, and analysed | 3-4 |
|  |  | (b) Give reasons for non-participation at each stage | Figure 1 |
|  |  | (c) Consider use of a flow diagram | Figure 1 |
| Descriptive data | 14 | (a) Give characteristics of study participants (eg demographic, clinical, social) and information on exposures and potential confounders | Tables 1 and 3 |
|  |  | (b) Indicate number of participants with missing data for each variable of interest | NA |
|  |  | (c) Cohort study—Summarise follow-up time (eg, average and total amount) | NA |
| Outcome data | 15 | Cohort study—Report numbers of outcome events or summary measures over time | Tables 2 and 4 |
| Main results | 16 | (a) Give unadjusted estimates and, if applicable, confounder-adjusted estimates and their precision (eg, 95% confidence interval). Make clear which confounders were adjusted for and why they were included | Tables 1-4 |
|  |  | (b) Report category boundaries when continuous variables were categorized | Tables 1-4 |
|  |  | (c) If relevant, consider translating estimates of relative risk into absolute risk for a meaningful time period | NA |
| Other analyses | 17 | Report other analyses done—eg analyses of subgroups and interactions, and sensitivity analyses | Tables S1-S5 |
| **Discussion** |  |  |  |
| Key results | 18 | Summarize key results with reference to study objectives | 4-6 |
| Limitations | 19 | Discuss limitations of the study, taking into account sources of potential bias or imprecision. Discuss both direction and magnitude of any potential bias | 9 |
| Interpretation | 20 | Give a cautious overall interpretation of results considering objectives, limitations, multiplicity of analyses, results from similar studies, and other relevant evidence | 4-9 |
| Generalisability | 21 | Discuss the generalisability (external validity) of the study results | 4-9 |
| **Other information** |  |  |  |
| Funding | 22 | Give the source of funding and the role of the funders for the present study and, if applicable, for the original study on which the present article is based | 10 |
